# Prediction of 3-year risk of diabetic kidney disease using machine learning based on electronic medical records

**DOI:** 10.1186/s12967-022-03339-1

**Published:** 2022-03-26

**Authors:** Zheyi Dong, Qian Wang, Yujing Ke, Weiguang Zhang, Quan Hong, Chao Liu, Xiaomin Liu, Jian Yang, Yue Xi, Jinlong Shi, Li Zhang, Ying Zheng, Qiang Lv, Yong Wang, Jie Wu, Xuefeng Sun, Guangyan Cai, Shen Qiao, Chengliang Yin, Shibin Su, Xiangmei Chen

**Affiliations:** 1 Department of Nephrology, First Medical Center of Chinese, PLA General Hospital, Nephrology Institute of the Chinese People’s Liberation Army, State Key Laboratory of Kidney Diseases, National Clinical Research Center for Kidney Diseases, Beijing Key Laboratory of Kidney Disease Research, No. 28 Fuxing Road, Beijing, 100853 China; 2 Medical Big Data Research Center, Medical Innovation Research Division of Chinese People’s Liberation, Army General Hospital, National Engineering Laboratory for Medical Big Data Application Technology, No. 28 Fuxing Road, Beijing, 100853 China

**Keywords:** Type 2 diabetes, Diabetic kidney disease, Electronic medical records, Machine learning, Light gradient boosting machine, Risk assessment

## Abstract

**Background:**

Established prediction models of Diabetic kidney disease (DKD) are limited to the analysis of clinical research data or general population data and do not consider hospital visits. Construct a 3-year diabetic kidney disease risk prediction model in patients with type 2 diabetes mellitus (T2DM) using machine learning, based on electronic medical records (EMR).

**Methods:**

Data from 816 patients (585 males) with T2DM and 3 years of follow-up at the PLA General Hospital. 46 medical characteristics that are readily available from EMR were used to develop prediction models based on seven machine learning algorithms (light gradient boosting machine [LightGBM], eXtreme gradient boosting, adaptive boosting, artificial neural network, decision tree, support vector machine, logistic regression). Model performance was evaluated using the area under the receiver operating characteristic curve (AUC). Shapley additive explanation (SHAP) was used to interpret the results of the best performing model.

**Results:**

The LightGBM model had the highest AUC (0.815, 95% CI 0.747–0.882). Recursive feature elimination with random forest and SHAP plot based on LightGBM showed that older patients with T2DM with high homocysteine (Hcy), poor glycemic control, low serum albumin (ALB), low estimated glomerular filtration rate (eGFR), and high bicarbonate had an increased risk of developing DKD over the next 3 years.

**Conclusions:**

This study constructed a 3-year DKD risk prediction model in patients with T2DM and normo-albuminuria using machine learning and EMR. The LightGBM model is a tool with potential to facilitate population management strategies for T2DM care in the EMR era.

## Background

Diabetic kidney disease (DKD) is a leading cause of end-stage renal disease (ESRD), cardiovascular (CV) disease, and all-cause morbidity and mortality in patients with diabetes [1]. Notably, diabetes and chronic kidney disease (CKD) are risk factors for severe COVID-19 infection and poor outcomes [2, 3]. Early identification of patients with diabetes who are at high risk for DKD will inform clinical decision-making. Understanding the risk factors that contribute to DKD and a precise DKD risk prediction model will allow early intervention in DKD and prevent its progression. Accurate prediction of DKD risk will drive the timely use of primary prevention strategies, and facilitate the identification of incident CKD in patients with diabetes before microalbuminuria appears. There remains an unmet clinical need for a precise predictive model of DKD risk that can be used to screen the large population of patients with diabetes and in management decisions.

Predictors of DKD risk include albumin excretion rate (AER), blood pressure, blood glucose, glomerular filtration rate (GFR), diabetic retinopathy, and plasma lipid levels. In the real-world setting, collecting longitudinal data from the large population of patients with diabetes is challenging [4]. In clinical practice, unselective screening for DKD is not cost-effective. The ability to predict DKD risk in individual patients with diabetes may be improved by a comprehensive and integrated evaluation of currently available clinical parameters.

Machine-learning of big medical data derived from electronic medical records (EMR) in the real-world setting is supporting physicians in their clinical diagnoses and management of asthma and life-style related diseases such as diabetes [5, 6]. Models that predict the risk of kidney failure (defined as replacement therapy-treated ESRD) among patients with CKD or the risk of ESRD in patients with DKD have been developed [7, 8]. To the author’s knowledge, there are no predictive models of DKD in patients with diabetes based on EMR constructed using machine learning.

A recent study revealed that the predictive power of a real-world data (RWD)–based model for diabetes-related CKD outperformed published algorithms based on data from clinical trials [9]. The objective of the present study was to construct a 3-year DKD risk prediction model in patients with type 2 diabetes mellitus (T2DM) and normo-albuminuria using machine learning, based on EMR. The model will augment physicians’ empirical judgments with rapid and precise predictions of DKD risk in patients with T2DM and normo-albuminuria and identify predictive risk factors for DKD among this patient population.

## Methods

### Data source

Data for this study were retrospectively derived from the EMR database at the People’s Liberation Army (PLA) General Hospital, the largest hospital in North China. The EMR database contains patient information and medical records from all hospital departments. The data set was de-identified and spanned from October 2008 to December 2019. This study was approved by the PLA General Hospital ethics committee (S2017-133-01) and conducted according to the guidelines of the Declaration of Helsinki.

### Study population

Patients diagnosed with T2DM, according to International Classification of Diseases (ICD)-10 codes, with 3 years of follow-up were eligible for this study. Exclusion criteria were: (1) aged < 18 years; (2) undergoing an invasive procedure; (3) presence of an acute infection; (4) presence of a malignancy; (5) or pregnancy.

At baseline, included patients had no evidence of DKD, defined as urinary albumin/creatinine ratio (UACR) > 30 mg/g, protein excretion rate > 150 mg/24 h, or urine dipstick test ≥ 1 + [10], or eGFR < 60 mL/min/1.73m^2^, calculated using the Chronic Kidney Disease Epidemiology Collaboration (CKD-EPI [11]) equation.

**Fig. 1:**
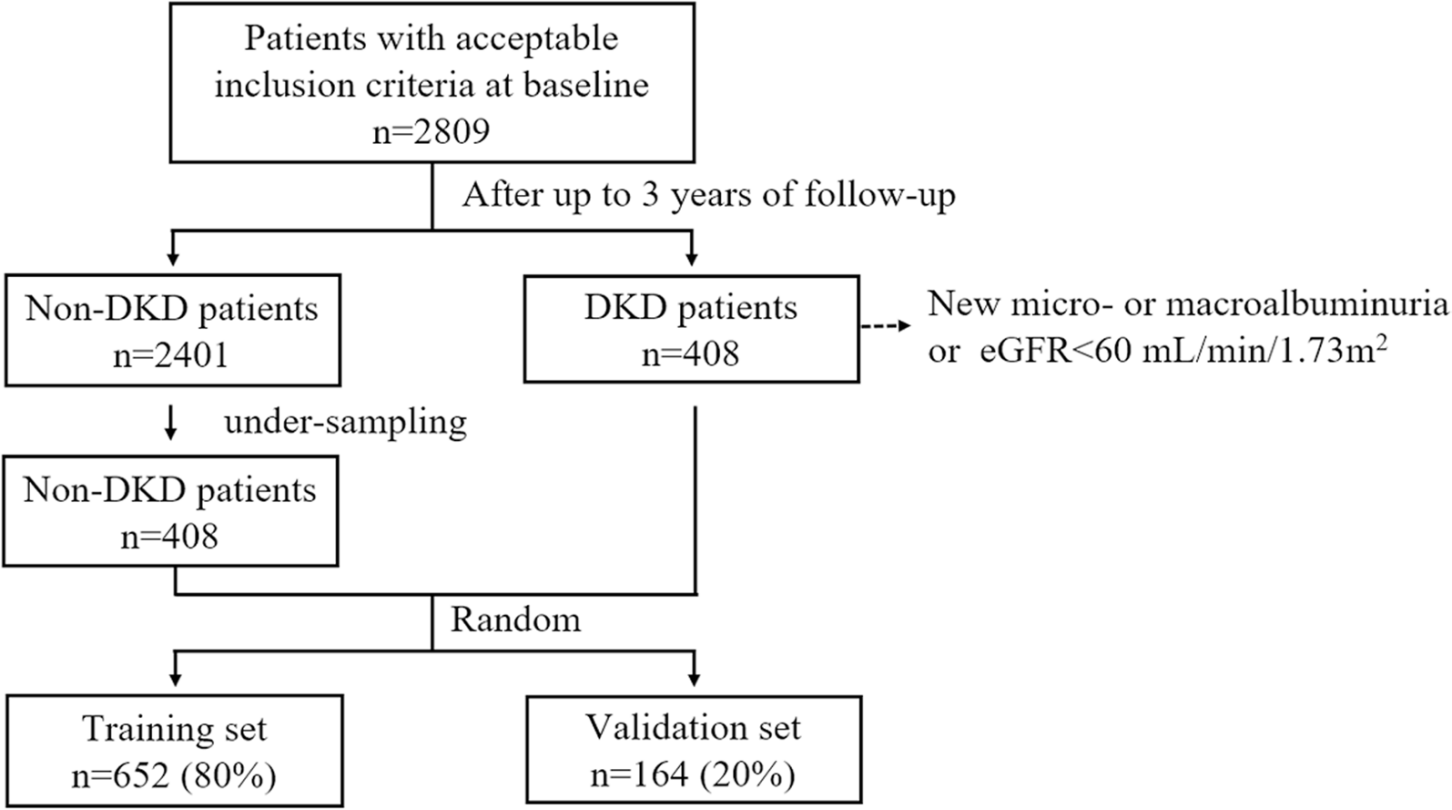
Flow diagram of patient selection. Non-DKD, no diabetic kidney disease; DKD, diabetic kidney disease; eGFR, estimated glomerular filtration rate

At the 3-year follow-up, included patients (n = 2809) were stratified according to the presence (n = 408) or absence (n = 2401) of DKD; under-sampling was used to balance the number of patients with or without DKD to 408 each [12]. Patients were randomly split 8:2 into a training set (n = 652) and a validation set (n = 164) using the Python package (Scikit-learn) [13] (Fig. 1).

### Candidate predictor variables

Patients’ demographic and clinical characteristics at baseline and the 3-year follow-up were recorded. Laboratory variables were derived from universally implemented tests. Comorbidities (presence or absence, number and type), including hypertension, cardiovascular disease, peripheral neuropathy, diabetic retinopathy and cerebrovascular disease, were diagnosed according to ICD-10 codes.

The risk prediction model was trained using 46 variables selected from medical reports and published literature, including sex, age, body weight and height, BMI, urine specific gravity (SG), urine red blood cell count (RBC), hemoglobin (Hb), hematocrit (Hct), mean corpuscular volume (MCV), mean corpuscular hemoglobin concentration (MCHC), white blood cell count (WBC), percent neutrophil granulocytes (N%), percent lymphocytes (L%), neutrophil to lymphocyte ratio (NLR), platelet count (PLT), mean platelet volume (MPV), activated partial thromboplastin time (APTT), plasma fibrinogen (FIB), random blood glucose (RBG), HbA1c, blood urea nitrogen (BUN), serum creatinine (SCR), serum uric acid (SUA), eGFR, total bilirubin (T-BiL), direct bilirubin (D-BiL), ALB, γ-glutamine transferase (GGT), total cholesterol (TC), triglyceride (TG), high-density lipoprotein (HDL), LDL, serum potassium (K), serum sodium (Na), calcium (Ca), phosphate (P), bicarbonate, and Hcy. Albuminuria was not used as a predictor, as patients had normal urinary protein excretion at baseline.

Variables with > 25% missing data were excluded. Missing values for included variables were imputed using the random forest (RF) method [14].

### Model development and evaluation

Seven machine learning algorithms implemented in the Python package 3.3.8: light gradient boosting machine (LightGBM) [15], eXtreme gradient boosting (XGBoost) [16], adaptive boosting (AdaBoost) [17], artificial neural network [18], decision tree [19], support vector machine (SVM) [20] and logistic regression [21], were used to identify the most informative variables for 3-year DKD risk prediction and develop models that predicted 3-year DKD risk as a binary outcome (presence or absence), according to the baseline values of the selected predictor variables.

LightGBM is a new member of the boosting family of algorithms, which is an accurate and efficient implementation of GBDT, similar to XGBoost. Both LightGBM and XGBoost take the negative gradient of the loss function to fit the residuals and find the optimal solution. Compared with XGBoost, LightGBM has faster training efficiency, lower memory, higher accuracy, and it can handle large-scale data and provide direct support of categories. The AdaBoost algorithm is a boosting method that combines multiple weak classifiers into a single strong classifier. The neural network model represents a (significant) enhancement of the logistic regression method. Decision tree models break down data sets into smaller subsets and develop an associated decision tree. The SVM algorithm is a binary classifier that maps input data into a high-dimensional feature space with a non-linear transformation. The logistic regression algorithm builds linear models with built in attribute selection.

A binary outcome for the prediction model was defined as the presence or absence of DKD. Every subset of data included the baseline values (at patients' first visit) for the predictor variables as well as DKD outcomes at 3 years of follow-up. Data were randomly allocated into separate training and validation data sets for each time window using the Python package (Scikit-learn) [13]. 80% of the data was used for training the model, and the remaining 20% was used to validate the model’s predictive performance.

### Statistical analysis

Analyses were conducted using Python version 3.8.3 and SPSS software (version 25.0; SPSS Inc., Chicago, IL, USA). Normally distributed continuous variables were compared using the student's t test. Non-normally distributed continuous variables were compared using the Wilcoxon rank sum test. Categorical variables were compared using the chi-square test. Tests were two-sided. A *P* value < 0.05 was considered statistically significant.

Performance of the predictive models generated by the seven machine learning algorithms were evaluated using the area under the receiver operating characteristic (ROC) curve (AUC), sensitivity, specificity, accuracy, and the F1 score ( 2* ( (precision*recall)/ (precision + recall)); range from 0 (worst score) to 1 (best score)) [22]. Shapley additive explanation (SHAP) was used to interpret the results of the best performing prediction model by computing the contribution of each variable to the prediction [23, 24]. SHAP values evaluate the importance of the output resulting from the inclusion of feature A for all combinations of features other than A [23].

## Results

### Patient characteristics

A total of 816 patients were included in this analysis. Of these, patients had a median age of 56 years (IQR, 48–66 years), and 585 (67.7%) patients were male. The incidence of at least one macrovascular or microvascular complication (hypertension, cardiovascular disease, cerebrovascular disease, diabetic retinopathy, diabetic peripheral neuropathy) was 52.6%. Baseline demographic and clinical characteristics of patients with or without DKD (n = 408 each) at the 3-year follow-up are shown in Table 1**.** At baseline, patients with no DKD at the 3-year follow-up were significantly older and had significantly higher eGFR, ALB, and Hb and significantly lower HbA1c, compared to patients with DKD at the 3-year follow-up.

**Table 1:** Baseline demographic and clinical characteristics of the included patients

Base line characteristics	All	non-DKD	DKD	*P value*
Patient population, n	816	408	408	
Male, n (%)	541 (66.3)	291 (71.3)	250 (61.3)	0.002
Age (years)	56.00 (48.25–65.00)	52.5 (47, 60)	61 (50, 71)	0.000
BMI (kg/m^2^)	26.03 (24.22, 28.61)	25.79 (24.46, 28.24)	26.30 (23.96, 29.06)	0.343
Hypertension (%)	349 (42.8)	157 (38.5)	192 (47.1)	0.013
Cardiovascular disease (%)	194 (23.8)	79 (19.4)	115 (28.2)	0.292
Cerebrovascular disease (%)	81 (9.9)	36 (8.8)	45 (11)	0.003
Peripheral neuropathy (%)	31 (3.8)	13 (3.2)	18 (4.4)	0.360
Diabetic retinopathy (%)	21 (2.6)	9 (2.2)	12 (2.9)	0.507
eGFR CKD-EPI (ml/min/1.73m^2^)	98.42 ± 18.63	103.25 ± 16.15	93.6 ± 19.69	0.000
SCR (μmol/L)	68.62 ± 14.06	67.13 ± 12.85	70.1 ± 15.05	0.003
BUN (mmol/L)	5.36 (4.5, 6.43)	5.22 (4.47, 6.16)	5.51 (4.53, 6.66)	0.004
SUA (μmol/L)	331.50 ± 91.85	336 ± 84.46	326.99 ± 98.58	0.161
HbA1c (%)	6.8 (6.3, 77.7)	6.6 (6.18, 7.30)	7.00 (6.41, 8.03)	0.000
ALB (g/L)	43.21 ± 4.04	43.89 ± 3.63	42.54 ± 4.31	0.000
TC (mmol/L)	4.41 (3.72, 5.23)	4.46 (3.85, 5.24)	4.33 (3.65, 5.23)	0.153
TG (mmol/L)	1.58 (1.11, 2.35)	1.69 (1.16, 2.42)	1.50 (1.03, 2.21)	0.019
HDL (mmol/L)	1.04 (0.89, 1.28)	1.03 (0.88, 1.26)	1.07 (0.90, 1.31)	0.107
LDL (mmol/L)	2.76 ± 0.91	2.82 ± 0.86	2.71 ± 0.95	0.09
K (mmol/L)	4.09 ± 0.38	4.07 ± 0.33	4.1 ± 0.42	0.372
Na (mmol/L)	142 (140, 143.3)	142.00 (140.80, 143.88)	141.60 (139.4, 143.00)	0.000
Ca (mmol/L)	2.29 ± 0.11	2.29 ± 0.10	2.28 ± 0.12	0.381
P (mmol/L)	1.18 ± 0.18	1.20 ± 0.18	1.17 ± 0.18	0.023
Bicarbonate (mmol/L)	26.13 (24.95, 27.5)	25.98 (24.9, 27.10)	26.23 (25.01, 27.78)	0.017
Hcy	12.59 (10.17, 15.45)	1175 (9.71, 14.16)	13.61 (10.74, 16.65)	0.000
Hb (g/L)	141.93 ± 18.8	145.51 ± 16.82	138.34 ± 19.98	0.000
NLR	1.84 (1.42, 2.44)	1.76 (1.35, 2.32)	1.97 (1.45, 2.61)	0.000
FIB (g/L)	3.09 (2.70, 3.54)	3.00 (2.65, 3.42)	3.18 (2.77, 3.69)	0.000

Values for continuous variables are expressed as mean ± standard deviation or median [interquartile range]; values for categorical data are given as number (percent). The *P* value represents comparison between non-DKD group and DKD group

Abbreviations and definitions: BMI, body mass index; eGFR, estimated glomerular filtration rate; SCR, serum creatinine; BUN, blood urea nitrogen; SUA, serum uric acid; ALB, serum albumin; TC, total cholesterol; TG, triglyceride; HDL. high-density lipoprotein; LDL, low-density lipoprotein; K, serum potassium; Na, serum sodium; Ca, calcium; P, phosphate; Hcy, homocysteine; Hb, hemoglobin; NLR, neutrophils to lymphocytes ratio; FIB, Plasma fibrinogen

### Feature selection

Recursive feature elimination (RFE) with RF was used to select variables as inputs for the 3-year DKD risk prediction model [25]. Ultimately, the 46 variables were reduced to 8 potential predictors of 3-year DKD risk. Five-fold cross validation combined with RF selected age, Hcy, HbA1c, BMI, Alb, eGFR and bicarbonate as the 7 most relevant variables. LDL was also included as it is a commonly cited risk factor for DKD.

### Model building and evaluation

**Fig. 2:**
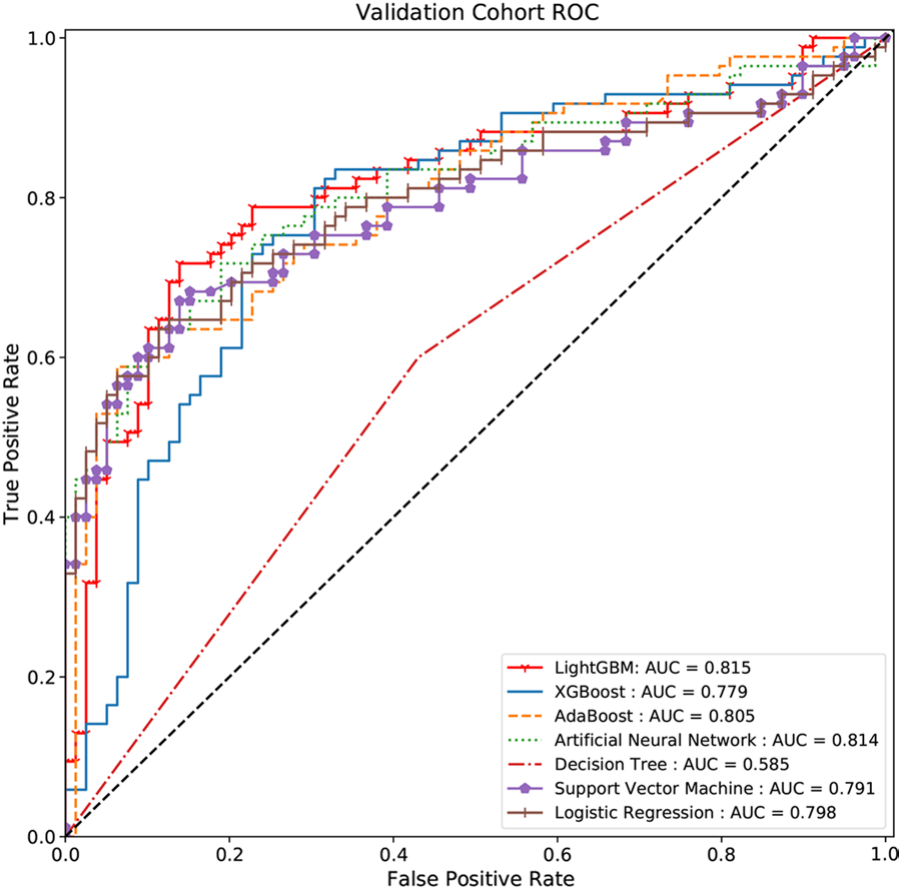
Evaluation of the seven machine learning algorithms based on the AUC of the ROC curve. AUC, area under the curve; ROC, receiver operating characteristic

The eight selected variables were used as inputs for the seven machine learning algorithms to predict 3-year DKD risk. Performance evaluation of the models generated by the seven machine learning algorithms is shown in Table 2. The LightGBM model had the highest AUC (0.815, 95% CI 0.747–0.882), sensitivity, positive predictive, and negative predictive values (Fig. 2). The decision tree had the lowest AUC value (0.579, 95% CI 0.503–0.655).

**Table 2:** Performance of the prediction models generated by the seven machine learning algorithms

Models	AUC	95% CI		SE (recall)	SP	AC	F1	PPV	NPV
		Lower bound	Upper bound						
LightGBM	0.815	0.747	0.882	0.741	0.797	0.768	0.768	0.797	0.741
XGBoost	0.779	0.706	0.853	0.682	0.785	0.732	0.725	0.773	0.697
AdaBoost	0.805	0.738	0.872	0.659	0.772	0.713	0.704	0.757	0.678
Artificial Neural Network	0.800	0.730	0.869	0.659	0.911	0.768	0.747	0.862	0.680
Decision Tree	0.579	0.503	0.655	0.576	0.595	0.579	0.587	0.598	0.603
Support Vector Machine	0.791	0.720	0.862	0.612	0.886	0.744	0.712	0.852	0.680
Logistic Regression	0.798	0.728	0.868	0.718	0.759	0.738	0.739	0.763	0.714

SE: sensitivity; SP: specificity; AC: accuracy; PPV: positive predictive value; NPV: negative predictive value

### Explanation of risk factor

**Fig. 3:**
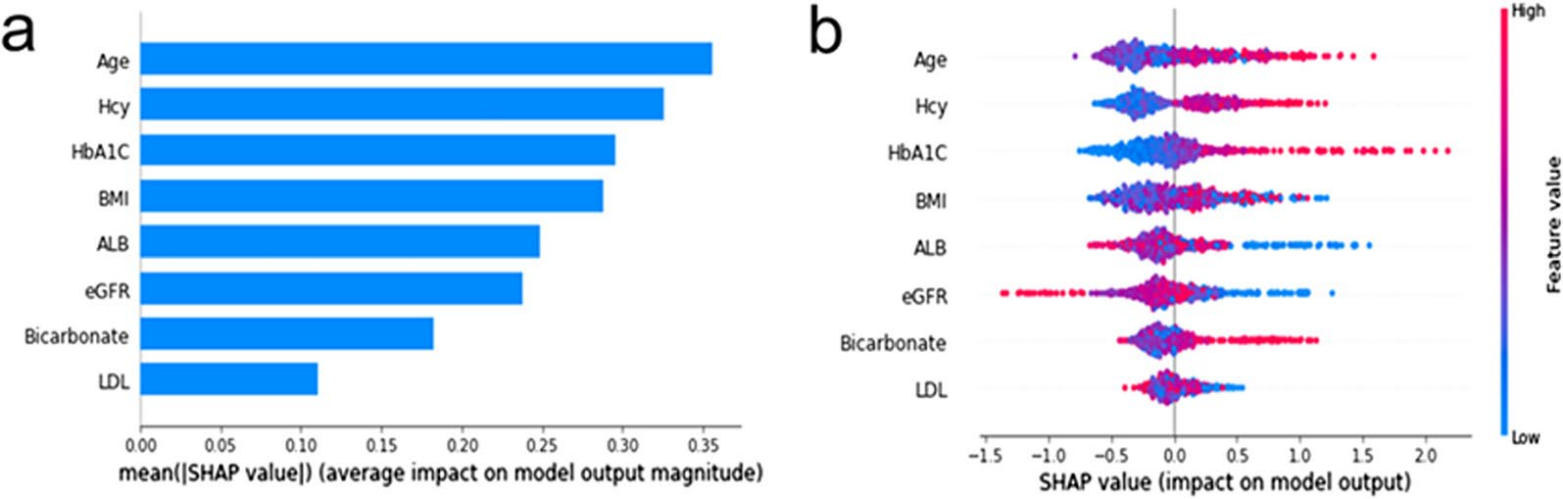
**a** Importance matrix plot of the LightGBM model, depicting the importance of each variable for predicting 3-year DKD risk in patients with T2DM and normo-albuminuria. **b** SHAP summary plot of the top 8 clinical features of the LightGBM model. There is one dot per patient per feature colored according to an attribution value, where red represents a higher value and blue represents a lower value. Hcy, homocysteine; BMI, body mass index; ALB, serum albumin; eGFR, estimated glomerular filtration rate; LDL, low-density lipoprotein

**Fig. 4:**
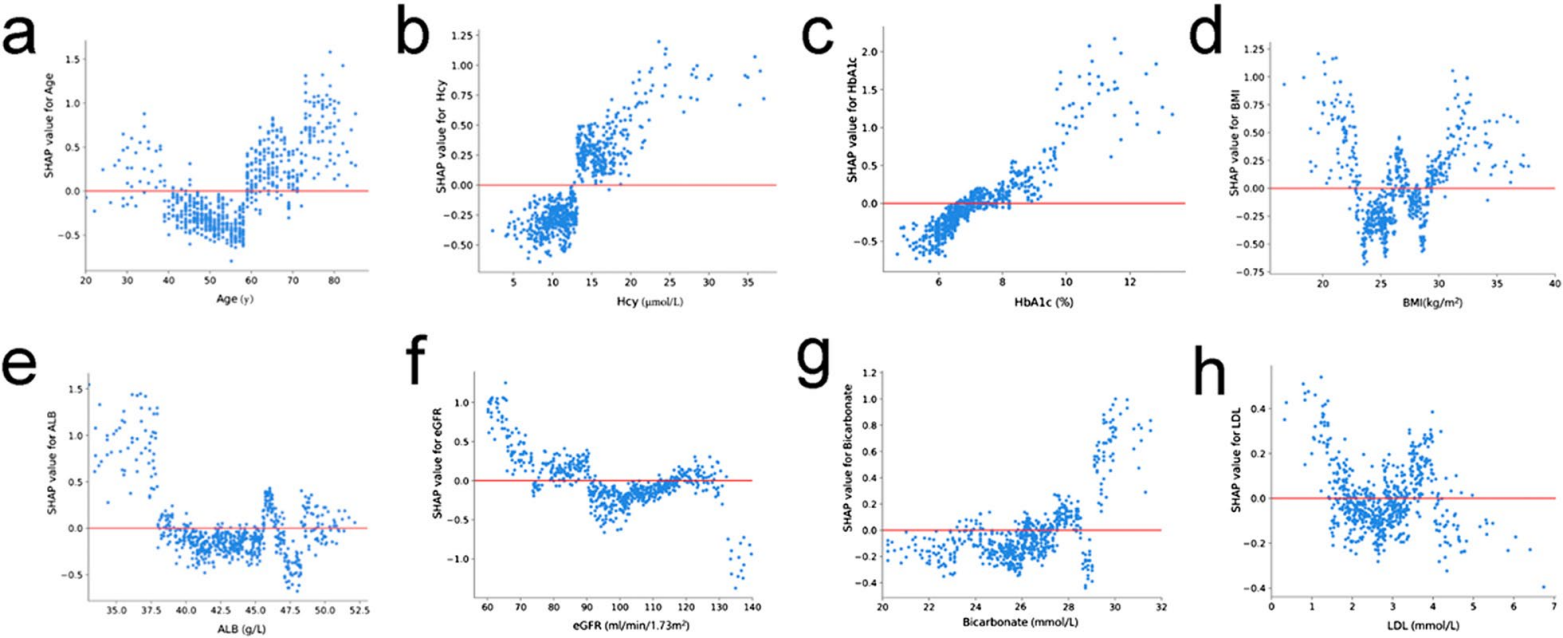
SHAP dependence plot of the LightGBM model, depicting how a single variable affects the prediction. SHAP values for specific features that exceed zero suggest an increased risk of DKD. Hcy, homocysteine; BMI, body mass index; eGFR, estimated glomerular filtration rate

SHAP was used to interpret the results of the LightGBM model by computing the contribution of each variable to the prediction. The importance matrix plot and SHAP summary plot for the LightGBM model are shown in Fig. 3, and the SHAP dependence plot for the LightGBM model is shown in Fig. 4.

The importance matrix plot ranked the variables contributing to 3-year DKD risk prediction from most to least important as patients’ baseline age, Hcy, HbA1c, BMI, Alb, eGFR, bicarbonate, and LDL (Fig. 3a). The SHAP summary plot (Fig. 3b) and SHAP dependence plot (Fig. 4) identified how each baseline variable influenced the outcome of DKD. On the SHAP summary plot, baseline variables with higher SHAP feature values increased the risk of developing DKD over the next 3 years. On the SHAP dependence plot, each dot represented a patient, such that the plot depicted how the attributed importance of a baseline variable changed with its value. SHAP values exceeding zero represented an increased risk of 3-year DKD. In general, older patients (Fig. 4a) with high Hcy (Fig. 4b), poor glycemic control (Fig. 4c), low Alb (Fig. 4e), low eGFR (Fig. 4f), and high bicarbonate (Fig. 4g) had an increased risk of developing DKD over the next 3 years. High or low BMI (Fig. 4d) and LDL (Fig. 4h) are risk factors for DKD progression.

### Applying the prediction model

**Fig. 5:**
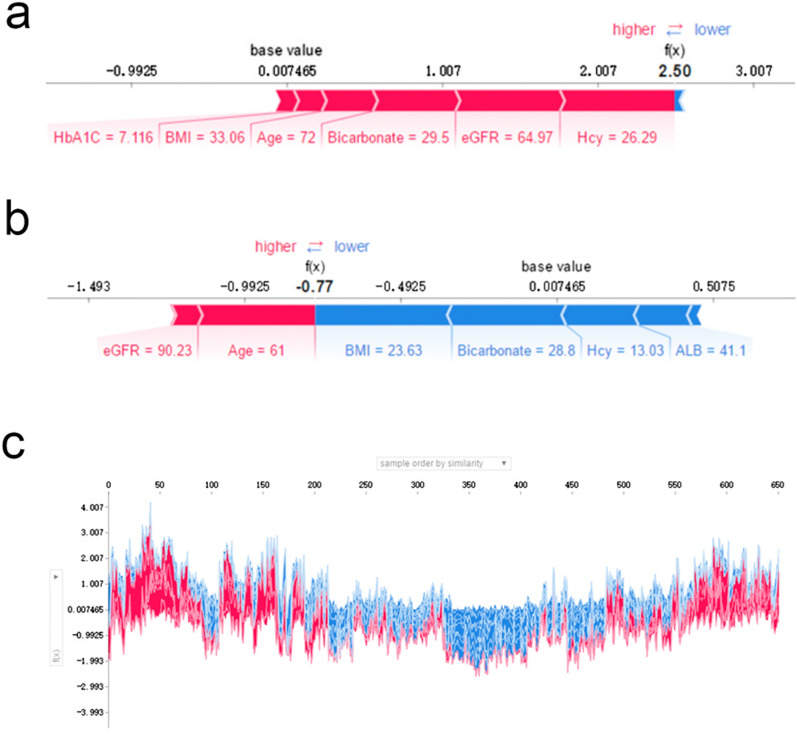
SHAP force plot for patients in the dataset at high (**a**) or low (**b**) risk of developing DKD; **c** SHAP values (global interpretation) for the training set. The abscissa represents each patient, and the ordinate represents the SHAP value. More red indicates a higher overall risk. Hcy, homocysteine; BMI, body mass index; eGFR, estimated glomerular filtration rate

SHAP force plots illustrate profiles of patients at high or low risk for developing an outcome and show how a predictive model based on EMR can facilitate individualized care planning. SHAP force plots for the LightGBM model are shown in Fig. 5.

In the study population, the risk of developing DKD over the next 3 years was 49.6%. Figure 5a shows a 72-year-old female patient with a predicted 92.4% [26] probability of developing DKD over the next 3 years. Addressing the modifiable risk factors of BMI, HbA1c, Hcy, eGFR, and bicarbonate may reduce this risk. Figure 5b shows a 61-year-old female patient with a lower risk profile; this patient had a predicted 31.7% [26] probability of developing DKD over the next 3 years. Figure 5c shows the risk of developing DKD over the next 3 years in the training set was 49.6%. Predictive values for each patient are listed in supplementary materials [27].

## Discussion

This study identified predictive risk factors for DKD and constructed a 3-year DKD risk prediction model in patients with T2DM and normo-albuminuria using machine learning and clinical variables easily extracted from EMR. The performance of predictive models generated by seven machine learning algorithms were compared. Findings showed the LightGBM model had the highest AUC, sensitivity, positive predictive, and negative predictive values. LightGBM is a high-performance gradient boosting framework [28, 29] that has been used for the prediction of undiagnosed T2DM, based on EMR [30]. To the author’s knowledge, this is the first published study to apply the LightGBM algorithm to predict the 3-year risk of DKD in patients with T2DM and normo-albuminuria who attended a hospital.

EMR has increased access to large amounts of patient data. This, combined with machine learning, is facilitating the development of sophisticated prediction models [31, 32]. Previous reports have presented machine learning techniques as black boxes, providing little information on how predictions have been made. This has hampered uptake by clinicians, who are reluctant to make medical diagnoses based on non-transparent decision-making. In this study, to facilitate interpretation of the decision process of the LightGBM algorithm, we used SHAP methodology to explain our predictions [33]. Baseline age, Hcy, HbA1c, BMI, Alb, eGFR, bicarbonate and LDL were selected as variables relevant for predicting 3-year DKD risk in patients with T2DM and normo-albuminuria. Previous studies have identified these as medically, socially, and economically important variables for quantifying the risk of CKD as a microvascular long-term complication of diabetes [34–36]. Consistent with this, our SHAP summary and dependence plots showed that baseline age, Hcy, HbA1c, Alb, eGFR and bicarbonate could distinguish patients at high or low 3-year risk of developing DKD. Specifically, older patients with high Hcy, poor glycemic control, low Alb, low eGFR, and high bicarbonate had a high 3-year risk of developing DKD. SHAP visualizations provide clinical insight and inform clinical decision-making, but highlight the complexity of predictive models. In this case, SHAP dependence plots revealed an increased 3-year risk of DKD in patients with T2DM and normo-albuminuria who had high or low eGFR, BMI or LDL.

Machine learning has confirmed that several biomarkers have prognostic use and may help investigators identify novel risk factors and provide insight into disease pathogenesis [37]. Ongoing research has identified multiple risk factors for DKD. In the present study, patients with T2DM aged > 60 years, eGFR < 90 ml/min/1.73m^2^, poor glycemic control, and high or low BMI had a high 3-year risk of developing DKD. Accordingly, older age was identified as a risk factor for DKD progression, independent of diabetes duration, in patients with T2DM [38]; a prospective observational cohort study of patients with T2DM followed for 10-years reported that albuminuria, older age, hypertension, insulin therapy, and lower baseline eGFR were independent predictors of annual eGFR decline [39]; and poor glycemic control and elevated BMI have been associated with the development and progression of DKD [40]. eGFR, glycemic control, and BMI are modifiable risk factors for DKD, such that the rational use of sodium/glucose cotransporter-2 inhibitors (SGLT2i) and other drugs in patients with T2DM may be beneficial. Interestingly, the present study also showed patients with T2DM, normo-albuminuria and an eGFR120-130 ml/min/1.73m^2^ (hyperfiltration) had a high 3-year risk of developing DKD. While more research is required, evidence suggests that glomerular or whole kidney hyperfiltration is a major contributing factor to the development of DKD in patients with type 1 or T2DM [41, 42]. Specifically, cohort studies with 3–18 years of follow-up showed that GFR declines more rapidly in patients with hyperfiltration at baseline compared to those with normal GFR [43].

The relationship between lipid profile and DKD is complex. Previous reports suggest dyslipidemia as a potential risk marker for DKD, but it is unclear which lipids or lipoproteins should be targeted for intervention [44]. In this study, LDL was included as a potential predictor of 3-year DKD risk, and it had a small impact on the output of the prediction model. Consistent with this, renal progression was significantly associated with LDL-cholesterol in patients with T1DM and normoalbuminuria followed for 8–9 years [44], substantiating experimental data and clinical studies that show targeted use of statins may represent a successful renoprotective strategy in diabetes [45, 46]. Irrespective of the association with DKD, dyslipidemia has a strong association with overall cardiovascular risk, making the control of dyslipidemia, especially LDL, essential for patients with diabetes. The benefits of pursuing lipid targets in patients without known cardiovascular disease are controversial [47]. In the present study, 23.8% of patients had cardiovascular disease at baseline, and SHAP dependence plots revealed patients with T2DM and high or low or LDL had an increased 3-year risk of DKD.

Homocysteine, Alb, and bicarbonate are not traditionally associated with increased risk for DKD. However, one study in Chinese patients with diabetes indicated a causal relationship between elevated circulating homocysteine levels and risk of DKD [48]; in hospitalized Han patients with T2DM, low serum Alb concentration was independently associated with diabetic retinopathy and DKD [30]; and serum albumin was identified as an important predictor of ESRD in patients with T2DM and DKD from three clinical trials (RENAAL [n = 1513], IDNT [n = 1715]and ALTITUDE [n = 8561]) using a feedforward neural network [40]. Bicarbonate may represent a novel risk factor for DKD [49]. Patients with diabetes with advanced renal failure show a lower prevalence or a less severe degree of metabolic acidosis [50], potentially through feedback control involving systemic acid–base status and hydrogen ion production that inhibits ketoacid anion production [51].

This study has several strengths. First, we used RWD derived from EMR, which is likely more representative of the diverse T2DM patient population than data derived from clinical trials. Second, among the other algorithms, the LightGBM model performed the best. LightGBM is a highly optimized gradient boosting decision tree algorithm that can incorporate multiple clinical variables Third, we identified risk factors that have not been traditionally associated with increased risk for DKD. Fourth, most studies have targeted patients with CKD and an eGFR < 60 ml/min/1.73m^2^ or ESRD [52]. We included patients with DKD presenting with new-onset micro- and macro- albuminuria. Fifth, our model can be used by clinicians and nurses as a visual approach to predict 3-year risk of DKD in patients with T2DM and normo-albuminuria, appropriately manage patients with T2DM and normo-albuminuria at high-risk for DKD and to target risk factors for DKD, thus informing the allocation of healthcare resources. Last, the model can be used as a screening tool for clinical trials. Enriching trials with patients at high 3-year risk of developing DKD may reduce sample sizes and lead to more efficient drug development programs.

This study was associated with some limitations. It was conducted at a single institution, included a small sample size, and the missing information (e.g., use of hypoglycemic drugs, history of diabetes, blood pressure) in our EMR-derived data represented a potential bias. However, we believe our rigorous methodology generated a robust predictive model of 3-year DKD risk in patients with T2DM and normo-albuminuria. External validation using another data set is required to establish stability in the performance of our prediction model.

## Conclusion

In conclusion, we identified baseline demographic and clinical variables as predictive risk factors for DKD and constructed a 3-year DKD risk prediction model in patients with T2DM and normo-albuminuria using machine learning and EMR. We established the LightGBM model as a tool with potential to facilitate population management strategies for T2DM care in the EMR era.

## Data Availability

The datasets used and/or analyzed during the current study are available from the corresponding author on reasonable request.

## Abbreviations

DKD: Diabetic kidney disease
T2DM: Type 2 diabetes mellitus
EMR: Electronic medical records
LightGBM: Light gradient boosting machine
AUC: Area under the receiver operating characteristic curve
SHAP: Shapley additive explanation
Hcy: Homocysteine
ALB: Albumin
eGFR: Estimated glomerular filtration rate
GFR: Glomerular filtration rate
ESRD: End-stage renal disease
CV: Cardiovascular
CKD: Chronic kidney disease
AER: Albumin excretion rate
RWD: Real-world data
ICD: International Classification of Diseases
UACR: Urinary albumin/creatinine ratio
CKD-EPI: Chronic Kidney Disease Epidemiology Collaboration
SG: Specific gravity
RBC: Red blood cell count
Hb: Hemoglobin
Hct: Hematocrit
MCV: Mean corpuscular volume
MCHC: Mean corpuscular hemoglobin concentration
WBC: White blood cell count
N%: Percent neutrophil granulocytes
L%: Ercent lymphocytes
NLR: Neutrophil to lymphocyte ratio
PLT: Platelet count
MPV: Mean platelet volume
APTT: Activated partial thromboplastin time
FIB: Plasma fibrinogen
RBG: Random blood glucose
BUN: Blood urea nitrogen
SCR: Serum creatinine
SUA: Serum uric acid
T-BiL: Total bilirubin
D-BiL: Direct bilirubin
GGT: γ-Glutamine transferase
TC: Total cholesterol
TG: Triglyceride
HDL: High-density lipoprotein
K: Serum potassium
N: A serum sodium
Ca: Calcium
P: Phosphate
XGBoost: EXtreme gradient boosting
AdaBoost: Adaptive boosting
SVM: Support vector machine
GBDT: Gradient Boosted Decision Tree
